# Novel statistical approaches to identify risk factors for soil-transmitted helminth infection in Timor-Leste

**DOI:** 10.1016/j.ijpara.2021.01.005

**Published:** 2021-08

**Authors:** Jessica Yi Han Aw, Naomi E. Clarke, Helen J. Mayfield, Colleen L. Lau, Alice Richardson, Susana Vaz Nery

**Affiliations:** a Research School of Population Health, Australian National University, Canberra, Australia; b Kirby Institute, University of New South Wales, Sydney, Australia; c Statistical Consulting Unit, Australian National University, Canberra, Australia

**Keywords:** Soil-transmitted helminths, Water, Sanitation and hygiene, Risk factors, Logistic regression, Recursive partitioning, Bayesian networks

## Abstract

Soil-transmitted helminths (STHs) are parasitic intestinal worms that infect almost a fifth of the global population. Sustainable control of STHs requires understanding the complex interaction of factors contributing to transmission. Identifying risk factors has mainly relied on logistic regression models where the underlying assumption of independence between variables is not always satisfied. Previously demonstrated risk factors including water, sanitation and hygiene (WASH) access and behaviours, and socioeconomic status are intrinsically linked. Similarly, environmental factors including climate, soil and land attributes are often strongly correlated. Alternative methods such as recursive partitioning and Bayesian networks can handle correlated variables, but there are no published studies comparing these methods with logistic regression in the context of STH risk factor analysis. Baseline cross-sectional data from school-aged children in the (S)WASH-D for Worms study were used to compare risk factors identified from modelling the same data using three different statistical techniques. Outcomes of interest were infection with *Ascaris* spp. and any hookworm species (*Necator americanus, Ancylostoma duodenale*, and *Ancylostoma ceylanicum*)*.* Mixed-effects logistic regression identified the fewest risk factors. Recursive partitioning identified the most WASH and demographic risk factors, while Bayesian networks identified the most environmental risk factors. Recursive partitioning produced classification trees that visualised potentially at-risk population sub-groups. Bayesian networks helped visualise relationships between variables and enabled interactive modelling of outcomes based on different scenarios for the predictor variables of interest. Model performance was similar across all techniques. Risk factors identified across all techniques were vegetation for *Ascaris* spp., and cleaning oneself with water after defecating for hookworm. This study adds to the limited body of evidence exploring alternative data modelling approaches in identifying risk factors for STH infections. Our findings suggest these approaches can provide novel insights for more robust interpretation.

## Highlights

• We compared logistic regression, recursive partitioning and Bayesian networks to identify risk factors for STH infection.
• Logistic regression identified fewest variables associated with STH infections compared with the two alternative methods.
• Recursive partitioning identified more demographic and WASH variables, and Bayesian networks more environmental variables.
• Model performance was similar across all three statistical techniques.
• Recursive partitioning can identify at-risk population subgroups, while Bayesian networks can run real-time scenarios.

## Graphical abstract

**Figure:**
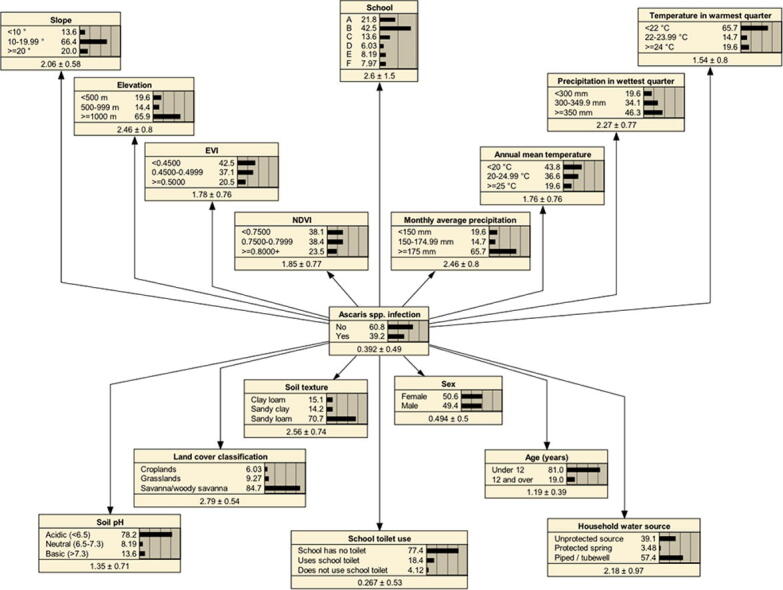

## 1 Introduction

Soil-transmitted helminths (STHs) are parasitic worms that include hookworms (*Necator americanus, Ancylostoma duodenale*, and *Anycylostoma ceylanicum*), roundworm (*Ascaris lumbricoides*), whipworm (*Trichuris trichiura*) and threadworm (*Strongyloides stercoralis*). Almost a fifth of the global population is infected with STHs, resulting in a burden of 3.8 million disability-adjusted life years (Kyu et al., 2018, World Health Organization and UNICEF, 2015). Long-term health consequences of STH infections in children include impaired growth and cognition, as well as malnutrition and iron-deficiency anaemia (Bethony et al., 2006).

STH transmission occurs due to contamination of soil, water sources and fresh produce with STH eggs that are released in faeces of infected individuals (WHO, 2020 https://www.who.int/news-room/fact-sheets/detail/soil-transmitted-helminth-infections).

Since STHs spend part of their life cycle in soil, environmental factors also play an important role in transmission. Previous studies have identified associations between STH infections and water, sanitation and hygiene (WASH), including both access and behaviours (Campbell et al., 2017, Freeman et al., 2017). Environmental factors such as vegetation, precipitation, temperature, elevation, land cover and soil attributes have also been shown to be associated with STH infections (Campbell et al., 2017, Wardell et al., 2017). However, specific WASH and environmental risk factors are not consistently identified across studies, even those conducted within similar contexts, impacting the robustness of evidence (Campbell et al., 2017, Freeman et al., 2017, Wardell et al., 2017).

Attempts to identify risk factors for STHs have been centred around the approach of logistic regression (LR) (Hosmer Jr et al., 2013). However, LR relies on the assumption of independence between predictor variables. This proves difficult in the STH context as many predictors are intrinsically associated, such as socioeconomic status (SES) and access to adequate WASH (Spratt et al., 2013, Ranganathan et al., 2017). Additionally, the assumption of independence between variables means potential causality cannot be explored (Nguefack-Tsague, 2011).

Novel statistical approaches enable data to be handled differently, potentially producing novel insights into risk factors. In health research, methods such as recursive partitioning (RP) and Bayesian networks (BNs) have mostly been applied in genomics and chronic disease contexts (Lemon et al., 2003, Needham et al., 2007). RP is a non-parametric approach that allows consideration of correlated data and is an attractive method for identifying at-risk population sub-groups (Lemon et al., 2003, Spratt et al., 2013, Gass et al., 2014). Only one study has utilised RP to explore the associations between WASH and STHs (Gass et al., 2014). More recently, studies have utilised RP to examine risk factors for other neglected tropical diseases (NTDs) including schistosomiasis and dengue fever (Gazzinelli et al., 2017, Ong et al., 2018). Few studies have compared RP with LR and no studies have done so in the context of STH or NTD risk factors (Lemon et al., 2003). BNs provide the potential to explore causality, while the other approaches do not (Nguefack-Tsague, 2011, Needham et al., 2007). While a small number of studies have explored BNs in the context of infectious diseases such as leptospirosis (Lau et al., 2017, Mayfield et al., 2018), none have focused on STH infections and none have compared findings with LR.

Sustainable control of STHs requires appropriate risk factor identification, so that appropriate community-based interventions can be developed (Montresor, 2012, World Health Organization, 2017). RP and BNs are promising alternatives to LR and the comparison between these techniques in the context of WASH and environmental factors for STH infections warrants investigation.

This study aimed to compare three different statistical approaches (LR, RP and BNs) in determining risk factors for STH infections. The specific objectives of the study were:

(i) Identify risk factors for STH infections using LR, RP and BNs.
(ii) Compare similarities and differences in the types of risk factors identified.
(iii) Qualitatively evaluate each technique to explain relationships between risk factors.

## 2 Materials and methods

### 2.1 WASH and STH infection data sources

This study is a secondary analysis of baseline data from the (S)WASH-D for Worms pilot study (Clarke et al., 2016, Clarke et al., 2018). The study took place in six primary schools in Aileu and Manufahi municipalities in Timor-Leste, a country where STHs are endemic (Martins and McMinn, 2012, Campbell et al., 2016), and where access to improved water and sanitation is poor (WHO, 2015; United Nations Development Program, 2018). Participants were children in grades 1–6 who attended those schools and had written informed consent from their parents or guardians. Study participants came from a total of 17 communities including the communities in which the schools were located, and neighbouring communities (Supplementary Table S1).

Data were collected in the form of questionnaires (conducted as interviews) and stool samples between April and June 2015 (Clarke et al., 2016, Clarke et al., 2018). Questionnaires were completed by both participants and their caregivers. Children responded to questions on personal WASH behaviours including handwashing, defecation and shoe-wearing practises. Caregivers answered questions on household water access, storage and treatment, and socio-economic characteristics. Additional questionnaires for the school principals asked questions about the schools’ sanitation facilities. SES was derived from principal component analysis of household income, animal ownership, house construction (wall and floor type), appliance ownership and vehicle ownership (Filmer and Pritchett, 2001, Campbell et al., 2016). Based on the number of eigenvalues above 1, four principal components were identified to produce a wealth score that was categorised into quintiles from 1 (poorest) to 5 (wealthiest) (Campbell et al., 2016). A full list of variables is provided in Supplementary Table S2.

Stool samples were collected from study participants at schools and sent to the QIMR Berghofer Medical Research Institute in Brisbane, Australia, for diagnostic analysis using real-time multiplex quantitative PCR (qPCR) to detect and quantify STH species (Inpankaew et al., 2014, Llewellyn et al., 2016). The primary outcomes assessed in this study were *Ascaris* spp*.* infection and any hookworm infection (*N. americanus* and *Ancylostoma* spp.).

### 2.2 Environmental data and processing

Environmental data were obtained at the community level. For the six communities in which study schools were located, we used GPS coordinates of the schools. These were collected during the field study. For the remaining 11 communities, we used coordinates representing the geographic centroid of the community (Statistics Timor-Leste, 2019a, Statistics Timor-Leste, 2019b). To ensure coverage of most households within each community, a 1 km buffer around each community coordinate was used when extracting environmental data. This buffer size was chosen with consideration of the sizes of communities in Timor-Leste and aerial maps were utilised to ensure that this buffer included a majority of households in each community.

Environmental data including climate, soil and land attributes were sourced from publicly available databases summarised in Table 1 (Loveland and Belward, 1997, Garcia et al., 1978, Belward et al., 1999; (Land Processes Distributed Active Archive Center and (LP DAAC), 2000, Weier and Herring, 2000, Hijmans et al., 2005, Didan et al., 2015, Wardell et al., 2017). Data were processed using ArcMap version 10.7 (Esri, Redlands, CA, USA). Slope was calculated from elevation data using the *slope* (spatial analyst) tool in ArcMap. Soil categorisations were determined by visual inspection of colour categorised maps. All other environmental data were processed using the *zonal statistics* (spatial analyst) tool in ArcMap. For land cover, the most common raster cell value within the buffer was used to classify each community. For temperature, precipitation, vegetation indexes (enhanced vegetation index (EVI) and normalised difference vegetation index (NDVI)), elevation and slope, the mean cell raster value was extracted. A range of temperature and precipitation variables were produced by calculating variations of monthly climate data, detailed in Supplementary Table S2 (Wardell et al., 2017).

**Table 1:** Summary of environmental data sources.

Environmental data type	Source	Temporal resolution	Spatial resolution
Temperature (°C)	WorldClim^a^	Monthly average precipitation from 1970 – 2000	1000 m
Maximum			
Minimum			
Mean			
Precipitation (cm)	WorldClim^a^	Monthly average precipitation from 1970 − 2000	1000 m
Maximum			
Minimum			
Mean			
Elevation per 100 m	ASTER GDEM^b^	30 January 2000–30 November 2013	30 m
Vegetation			
	Terra and Aqua MODIS^b^	1 April 2015–30 June 2015	250 m
Enhanced vegetation index (EVI)			
Normalised difference vegetation index (NDVI)			
Soil pH	Os Solos De Timor^c^	1960s	N/A
Soil Texture			
Land cover	MODIS^b^	2015	250 m

ASTER, Advanced Spaceborne Thermal Emission and Reflection Radiometer; GDEM, Global Digital Elevation Model version 3; MODIS, Moderate Resolution Imaging Spectroradiometer.

a Worldclim version 2.0 is a database providing monthly averaged climate data for mean, minimum and maximum precipitation and temperature.

b Elevation, vegetation and land cover data were sourced from the NASA EOSDIS Land Processes Distributed Active Archive Centre database (LP DAAC) using the Application for Extracting and Exploring Analysis Ready Samples (AppEEARS). We used data from the ASTER GDEM and Terra and Aqua MODIS satellites.

c Soil texture and pH data were derived from ‘Os Solos de Timor’, an extensive soil study of Timor-Leste conducted in the 1960s.

### 2.3 Statistical analysis

#### 2.3.1 Descriptive statistics

Descriptive analysis was conducted using Stata version 15 (College Station, TX, USA). Point prevalence and 95% confidence intervals (CIs) were calculated for all categorical variables using the *proportion* function in Stata. For continuous variables, mean values, S.D.s) and their 95% CIs were calculated using the *mean* function in Stata.

#### 2.3.2 Logistic regression

Generalised linear mixed models were constructed with school and community as nested random effects (communities nested within schools), and age and sex as fixed effects using the *meglm* function in Stata. Bernoulli logistic regression was used to produce an odds ratio for each variable. First, univariable analysis was conducted and variables retained if *P* < 0.2 (Aw et al., 2019, Vaz Nery et al., 2019). A two-stage approach was then used to build multivariable models (Aw et al., 2019, Vaz Nery et al., 2019). Retained variables were grouped into “within-domain” multivariable models adjusted for age and sex, for each of the following domains: demographic, individual hygiene, individual sanitation, school sanitation, household sanitation, household water and household socioeconomic variables. Prior to finalising within-domain models, a collinearity check was conducted using the *collin* function in Stata. Collinear variables were removed individually from within-domain models and tested for Akaike Information Criterion (AIC), with the lowest AIC determining which variable to remove. This process was repeated until all variables had a Variance Inflation Factor (VIF) < 5.0. Variables with *P <* 0.1 in “within-domain” models were retained for a full multivariable model adjusting for age and sex. Backward stepwise regression was used to produce the final multivariable model including only age, sex and variables with *P <* 0.05.

To examine model performance, data were first divided into three groups: each of the two largest schools, and all other schools combined. This was done because a much higher infection prevalence was observed in the two largest schools. Using these strata, the data were randomly partitioned 70:30. Coefficients for the variables in the final multivariable models were produced using data from 70% of the participants. Probability of infection was predicted in the remaining 30% of participants using the *predict* function in Stata. Participants were classified as infected if the predicted probability of infection was greater than 0.5. The area under the curve (AUC) was calculated using the *roctab* function in Stata.

#### 2.3.3 Recursive partitioning

The RP approach repeatedly partitions data into binary groups where subsequent groups are more likely to have the same outcome response (Strobl et al., 2009). The algorithm allows for different patterns after each partition. RP classification trees were produced for each outcome using the *rpart* package version 4.1.15 in R version 3.6.3.

Data were partitioned in the same way as for LR. Classification tree models were built using data from 70% of participants. The RP models underwent 10 cross-validations (on the 70% of data) which were averaged to produce complexity parameter (CP) values with associated cross-validation errors (x-errors). Over-fitting is a common problem with RP models and to address this, each classification tree was pruned (Gass et al., 2014). Typically, the final number of nodes (independent variables) of a tree is based on the CP value with the smallest x-error, where adding an additional node will no longer provide better classification (Therneau and Atkinson, 1997). In instances where the smallest x-error corresponded to a CP value for one or two nodes, the model was “lightly” pruned by the next smallest x-error that corresponded to a meaningful number of nodes. R does not allow “light” pruning if there is no substantial difference in performance compared with the unpruned model (Therneau and Atkinson, 1997). The remaining 30% of data were used to predict whether participants would be classified as infected or not. The AUC was calculated using the *pROC* package in R Studio.

#### 2.3.4 Bayesian networks

BNs consist of two main components: (i) directed acrylic graphs which contain nodes representing variables and arrows between nodes defining dependency; and (ii) node probability tables (NPTs) (Fenton and Neil, 2012). A child node is one that is conditionally dependent on one or more parent nodes. A conditional probability table (CPT) is the NPT of a child node. For feasible interpretation of CPTs, all continuous variables were discretised into three categories where data could be grouped in even intervals with sufficient sample size in each category (Supplementary Table S2) (Netica Software Corp., 2020). Since temperature and precipitation variables were variations of the same averaged monthly data, only mean annual temperature, mean monthly precipitation and one other temperature and precipitation variable (selected based on highest variance reduction) were included. For *Ascaris* spp*.*, precipitation in the wettest quarter was removed as most of its variance reduction was explained by the school variable. For consistency, data were categorised in the same way described for LR.

Firstly, naïve networks were built to examine the independent relationship between each variable and the outcome. Variance reduction of all variables was ranked to identify the most influential variables, which would be included in the final model. Variables with less than 0.5% variance reduction were automatically removed from the model. Next, the variable contributing the next lowest variance reduction was removed, and the model was retrained using data from 70% of participants and tested with the remaining 30%, with an AUC calculated. Variables were iteratively removed, model re-trained and tested until there was a noticeable decrease in AUC performance, at which point only school, age and sex variables remained for consistency with LR. The final combination of variables was selected based on the iteration with the highest AUC.

The final variables retained in the respective naïve models for *Ascaris* spp. and hookworm were used to learn tree augmented naïve (TAN) networks. TAN networks automatically model relationships between variables with each variable having at most one parent node in addition to the target node (Friedman et al., 1997). Scenario analyses were conducted on each TAN model where the probability of a particular response in variables of interest (WASH access, behaviours and SES) was set to 100%. Estimated infection prevalence was then compared with the infection prevalence in the prior state.

Expert structured models were built based on existing literature on risk factors for *Ascaris* spp. and hookworm (Strunz et al., 2014, Campbell et al., 2017, Freeman et al., 2017, Wardell et al., 2017). Due to limited examples in the data to sufficiently represent combinations of responses, a limit of four parent nodes were linked to each outcome. Variables with the lowest variance reduction were removed (e.g. handwashing after food contact and household water source).

To examine model performance, 100 trials were conducted on all networks, training each on a new random sample of 70% of data and testing on a new random sample of 30% data. BN analysis was conducted on Netica (Norsys Software Corp, Vancouver, BC, Canada).

For all models across all techniques, sensitivity and specificity were also calculated. By adding sensitivity and specificity and subtracting the value of 1, a true skill statistic (TSS) was calculated where values greater than zero indicate that model performance was better than random classification (Somodi et al., 2017).

## 3 Results

The study population consisted of 464 participants who completed a questionnaire and provided a stool sample. Participant characteristics are summarised in Supplementary Table S3. Overall *Ascaris* spp*.* prevalence was 39.2% (95% CI: 15.1–70.1) and hookworm prevalence was 14.7% (95% CI 0.4–39.2). Nearly all hookworm infections (94%) were caused by *N. americanus.* The mean participant age was 9 years (S.D. 2.5) and 49.4% (95% CI: 44.3–54.4) were male. Over two-thirds of participants (67.6%, 95% CI: 42.8–85.4) reported having a household latrine; however, 57.8% (95% CI: 48.7–66.3) of participants practised open defecation. A protected household water source using the WHO/UNICEF Joint Monitoring Program definition (WHO, 2015) was reported by 60.9% (95% CI: 40.7–77.9) of participants. Hand hygiene behaviour was variable with 33.8% (95% CI: 25.2–43.8) of participants reporting handwashing before food contact and 56.0% (95% CI: 39.2–71.6) reporting handwashing after defecating.

### 3.1 Environmental characteristics

Environmental characteristics are summarised in Supplementary Table S4. Average monthly precipitation ranged from 103 to 105 mm while annual mean temperature ranged from 18.3 to 26.3 °C. NDVI and EVI generally indicated tropical vegetation. Savanna was the most predominant land cover type. Elevation between communities varied from sea level to 1515 metres. Acidic soil type (pH 5.5–6.49) was most common.

### 3.2 Logistic regression

Various WASH, demographic and environmental variables were significant at *P* < 0.2 in univariable analyses for *Ascaris* spp*.* and hookworm infection (Supplementary Tables S5 and S6). Results of the final multivariable models for *Ascaris* spp*.* and hookworm are presented in Table 2, Table 3, respectively.

**Table 2:** Results of multivariable logistic regression for *Ascaris* spp.

Covariate	*n*/mean^a^	% infected/S.D.^a^	aOR	95% CI	*P* value
Age (years)^b^	9	2.5	1.02	0.93–1.11	0.714
*Sex*					
Female	235	41.3	Ref		
Male	229	37.1	0.83	0.53–1.31	0.432
*School toilet use*					
School does not have toilet	357	34.7	Ref		
Does not use school toilet	85	65.9	**2.35**	**1.27–4.35**	**0.007**
Uses school toilet	19	5.2	0.27	0.03–2.52	0.248
*Household water is treated by boiling*					
Yes	385	40.5	**0.28**	**0.12**–**0.64**	**0.003**
No	74	29.7	Ref		
Mean temperature in coldest month (°C)^c^	14.6	3.1	**0.68**	**0.47–0.83**	**<0.001**
NDVI^d^	0.75	0.05	**1.10**	**1.01–1.20**	**0.027**
*Random effects variance (95% CI)*					
School	0.61(0.10–3.62)				
Village	<0.001				

*n*, sample size; % infected, proportion infected; aOR, adjusted odds ratio; 95% CI, 95% confidence interval; Ref, reference category; NDVI, normalised difference vegetation index. Bold text indicates *P* < 0.05.

The model includes 456 participants from six schools and 17 communities, with school and community as random effects. Values presented are produced from 100% of the data.

a Age, mean temperature in coldest month and NDVI are all continuous variables so the relevant mean and S.D. are presented instead of sample size and % infected, respectively.

b aOR for age refers to a 1 year increase in age.

c Mean temperature in coldest month refers to the average mean temperature in July from 1970-2000.

d NDVI is an index quantifying vegetation and is measured between −1 to 1 with values closer to 1 indicating more vegetation. aOR for NDVI refers to an 0.01 unit increase in NDVI as the variable was transformed by a factor of 100.

**Table 3:** Results of multivariable logistic regression for hookworm.

Covariate	n/mean^a^	% infected/S.D.^a^	aOR	95% CI	*P* value
Age (years)^b^	9	2.5	**1.24**	**1.10–1.40**	**<0.001**
*Sex*					
Female	235	13.2	Ref		
Male	229	16.2	1.23	0.68**–**2.22	0.324
*Cleaning oneself with water after defecating*					
Yes	222	6.7	**0.37**	**0.18–0.76**	**0.002**
No	235	22.1	Ref		
*Socioeconomic quintile*					
Quintile 1 or 2 (poorest)	119	27.7	Ref		
Quintile 3	105	7.6	**0.19**	**0.08–0.47**	**<0.001**
Quintile 4	114	13.2	**0.32**	**0.14–0.69**	**0.004**
Quintile 5	126	9.5	0.42	0.17–1.02	0.056
*Random effects variance (95% CI)*					
School	0.76(0.19–3.06)				
Village	<0.0001				

*n*, sample size; % infected/S.D., proportion infected or standard deviation; aOR, adjusted odds ratio; 95% CI, 95% confidence interval; Ref, reference category. Bold text indicates *P* < 0.05.

The model includes 456 participants from six schools and 17 communities, with school and community as random effects. Values presented are produced from 100% of the data.

a Age is a continuous variable so mean and S.D. were provided instead of sample size and % infected, respectively.

b aOR for age refers to a 1 year increase in age.

For *Ascaris* spp*.*, odds of infection were significantly increased in children who had access to, but did not use, their school toilet compared with children whose school had no toilet (adjusted odds ratio (aOR) 2.35, 95% CI: 1.27–4.35) (Table 2). Children from households where water was treated by boiling had significantly decreased odds of infection (aOR 0.28, 95% CI: 0.12–0.62). Higher temperature in the coldest month (July) was associated with decreased odds of infection (aOR 0.68, 95% CI: 0.47–0.83), while a higher NDVI or denser vegetation was associated with increased odds of infection (aOR 1.10, 95% CI: 1.01–1.20). In a sensitivity analysis excluding environmental variables, the same non-environmental variables remained significant (Supplementary Table S7).

For hookworm, odds of infection were significantly higher with increasing age. Cleaning oneself with water after defecating was associated with significantly decreased odds of infection (aOR 0.37, 95% CI: 0.18–0.76*)*. Belonging to socioeconomic quintiles 3 or 4 both decreased odds of infection compared with quintile 1 (poorest) or 2. No environmental variables were included in the final model for hookworm.

For both *Ascaris* spp*.* and hookworm outcomes, random effects variance indicated some variation at the school level but very little variation at the community level (Table 2, Table 3).

Model diagnostics comparing predicted and actual classification of infection revealed the *Ascaris* spp. model had a sensitivity of 53.7%, specificity of 83.3%, TSS of 0.370 and AUC of 0.738. The hookworm model had a sensitivity of 15.4%, specificity of 98.2%, TSS of 0.136 and AUC of 0.757.

### 3.3 Recursive partitioning

**Fig. 1:**
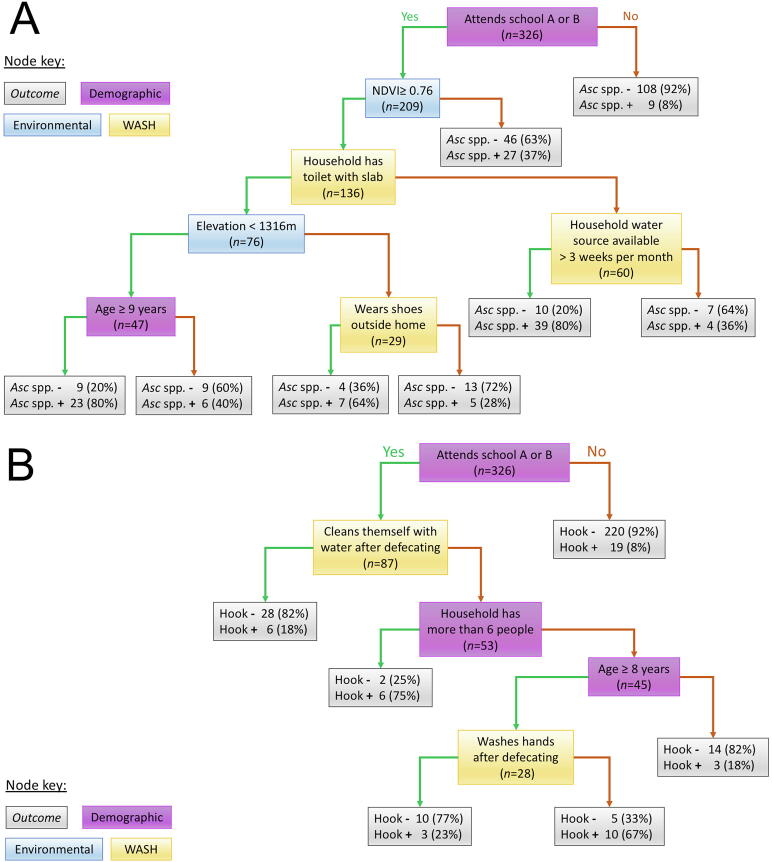
Classification trees for (A) *Ascaris* spp. and (B) hookworm infections from recursive partitioning. Each node represents an independent variable by which the model partitions the sample. Nodes are categorised into four colours broadly representing demographic, environmental and water, sanitation and hygiene (WASH) variables as well as the outcome node. The outcome node displays the number and percentage of participants infected and not infected with *Ascaris* spp*.* denoted as ‘*Asc.* spp. *+’* and ‘*Asc* spp. *–*’, and hookworm denoted as ‘Hook *+’* and ‘Hook *–’*. A green arrow from the left of a parent node indicates that participants in the subsequent node fit the criteria described in the parent node. A red arrow from the right of a parent node indicates that the participants in the subsequent node did not fit the criteria described in the parent node. Normalised difference vegetation index (NDVI) in (A) refers to a vegetation index taking values between −1 and 1 (more vegetation).

Classification trees for *Ascaris* spp*.* and hookworm identified a range of demographic WASH and environmental variables as influential (Fig. 1). The final tree for *Ascaris* spp*.* was lightly pruned to seven nodes using a CP of 0.17 as complete pruning based on lowest x-error resulted in a tree with two nodes. A total of eight outcomes were identified. The variables in order of conditionality were school, NDVI, household toilet structure, elevation and household water source availability, followed by age and wearing shoes outside the home.

For hookworm, complete pruning resulted in zero nodes so an unpruned final model with five nodes and a CP of 0.01 was used. Six outcomes were identified. The variables in order of conditionality were school, cleaning oneself with water after defecating, household with more than six people, age and handwashing after defecating.

**Supplementary figure S1:**
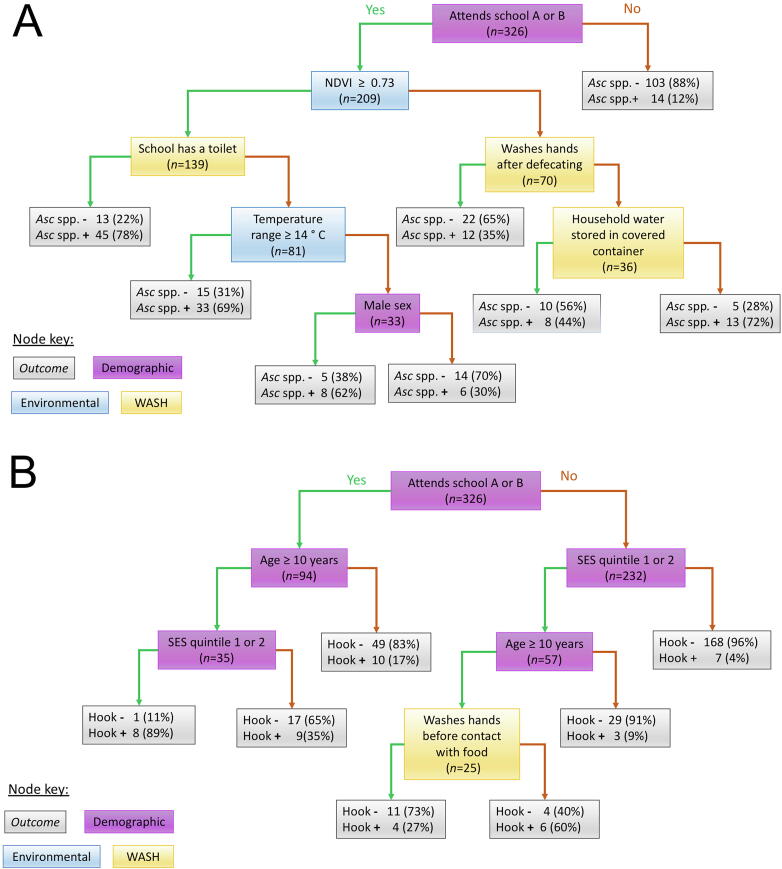
First sensitivity analysis for (A) Ascaris spp. and (B) hookworm classification trees from recursive partitioning. Each node represents an independent variable by which the model partitions the sample. Nodes are categorised into four colours broadly representing demographic, environmental and water, sanitation and hygiene (WASH) variables as well as the outcome node. The outcome node displays the number and percentage of participants infected and not infected with Ascaris spp. denoted as ‘Asc. spp. +’ and ‘Asc spp. –’, and hookworm denoted as ‘Hook +’ and ‘Hook –’. A green arrow from the left of a parent node indicates that individuals in the subsequent node fit the criteria described in the parent node. A red arrow from the right of a parent node indicates that the individuals in the subsequent node did not fit the criteria described in the parent node. Temperature range in (A) refers to the difference between the mean temperatures in the hottest (November) and coldest (July) months; normalised difference vegetation index (NDVI) refers to a vegetation index taking values between -1 and 1 (more vegetation). SES, socioeconomic status.

**Supplementary figure S2:**
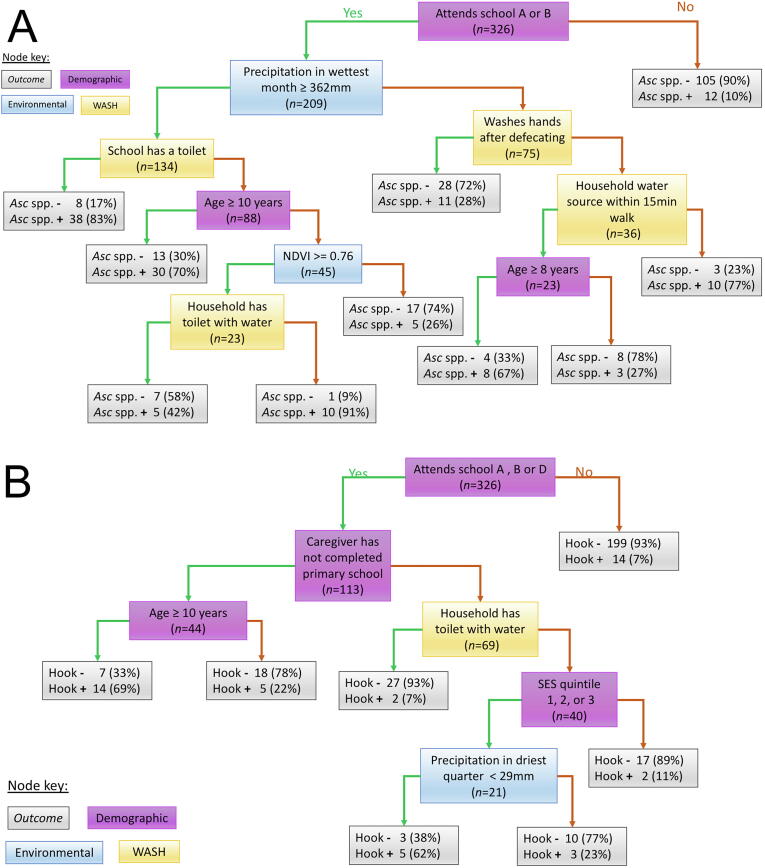
Second sensitivity analysis for (A) Ascaris spp. and (B) hookworm classification trees from recursive partitioning. Each node represents an independent variable by which the model partitions the sample. Nodes are categorised into four colours broadly representing demographic, environmental and water, sanitation and hygiene (WASH) variables as well as the outcome node. The outcome node displays the number and percentage infected and not infected with Ascaris spp. denoted as ‘Asc spp. +’ and ‘Asc spp. –’, and hookworm denoted as ‘Hook +’ and ‘Hook –’. A green arrow from the left of a parent node indicates that individuals in the subsequent node fit the criteria described in the parent node. A red arrow from the right of a parent node indicates that the individuals in the subsequent node did not fit the criteria described in the parent node. Normalised difference vegetation index (NDVI) in (A) refers to a vegetation index taking values between -1 and 1 (more vegetation). Precipitation in wettest month in (A) refers to February and precipitation in driest quarter in (B) refers to August-October. SES, socioeconomic status.

Model diagnostics revealed that the *Ascaris* spp. model had a sensitivity of 38.7%, specificity of 89.5%, TSS of 0.282 and AUC of 0.760. The hookworm model had a sensitivity of 23.8%, specificity of 90.6%, TSS of 0.144 and AUC of 0.691. A sensitivity analysis on an additional two different partitioned datasets revealed differences in the variables identified but the proportion of environmental, WASH and demographic variables remained relatively consistent (Supplementary Figs. S1, S2).

### 3.4 Bayesian networks

**Fig. 2:**
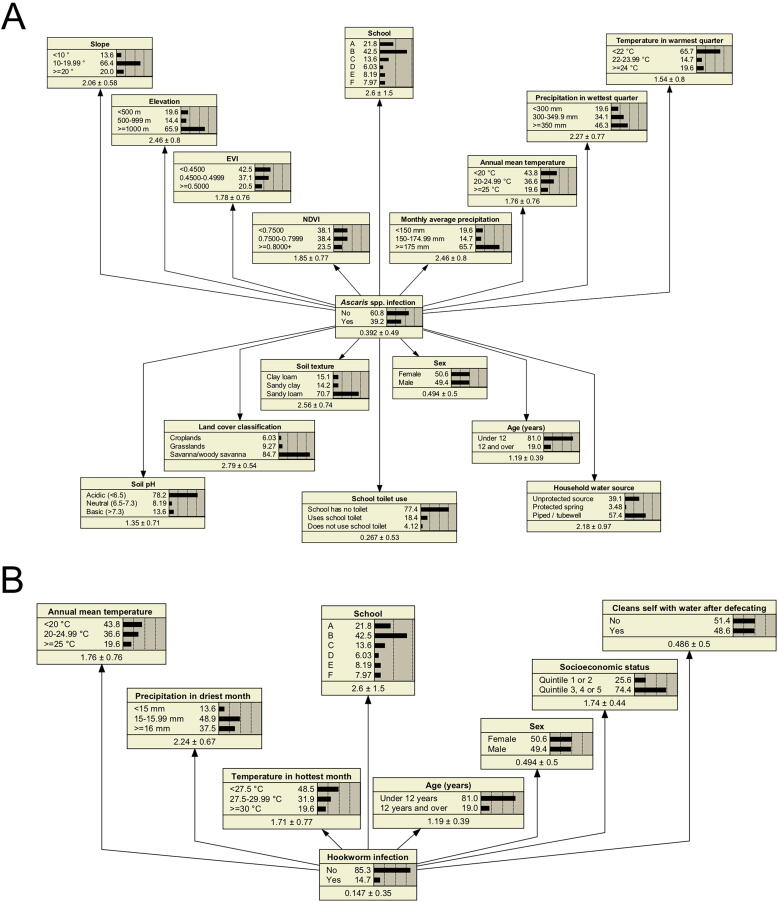
Naïve networks for (A) *Ascaris* spp. and (B) hookworm infections. The naive model assumes all variables are independent, shown with unidirectional arrows from the outcome to each predictor variable. Number values in each node box represent respective variable response probabilities based on 100% of data. Temperature and precipitation data are averaged from 1970 to 2000. Temperature in warmest quarter in (A), refers to October-December; precipitation in wettest quarter refers to December-February. Temperature in the hottest month in (B), refers to November; precipitation in driest month refers to September. Enhanced vegetation index (EVI) and normalised difference vegetation index (NDVI) in (A) refer to vegetation indexes taking values between −1 and 1 (more vegetation). Age and sex were included for consistency with logistic regression (LR) models but were not significant during variable selection.

**Fig. 3:**
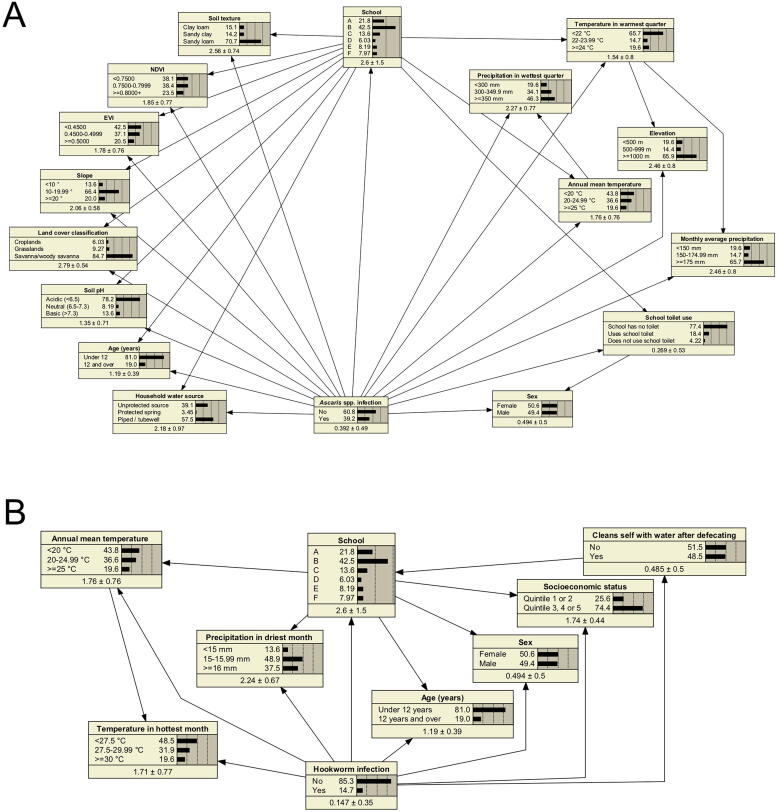
Tree augmented naïve (TAN) networks for (A) *Ascaris* spp. and (B) hookworm infections. The Tree Augmented Naïve (TAN) model depicts relationships between variables, with any variable being connected to the outcome and one other variable. Number values in each node box represent respective variable response probabilities based on 100% of data. Temperature and precipitation data are averaged from 1970 to 2000. Temperature in warmest quarter in (A), refers to October-December; precipitation in wettest quarter refers to December-February. Temperature in the hottest month in (B) refers to November; precipitation in driest month refers to September. Enhanced vegetation index (EVI) and normalised difference vegetation index (NDVI) in (A) refer to vegetation indexes taking values between −1 and 1 (more vegetation). Age and sex were included for consistency with logistic regression (LR) models but were not significant during variable selection.

Naïve, TAN and expert structured networks were constructed for *Ascaris* spp*.* and hookworm. In the *Ascaris* spp*.* naïve network, a total of 16 independent variables were included, consisting of environmental variables, household water source, school toilet use, school, age and sex (Fig. 2A). A TAN network was constructed based on the same variables, revealing how school was correlated with almost all variables, with the exception of a few environmental variables that were correlated with one another (Fig. 3A).

For hookworm, the initial naïve model with the highest AUC had 0% sensitivity. The variables for annual mean temperature and precipitation in driest month were added back into the model to improve the sensitivity performance, selected based on next highest variance reductions. Additional variables included in the hookworm naïve network were cleaning oneself with water after defecating, SES, school, age and sex (Fig. 2B). The TAN network revealed that all variables were correlated with school (Fig. 3B).

Scenario analyses were conducted on each TAN network to explore how changing the probability of a response category within a variable would impact the predicted outcome (Table 4). In the scenario where all participants had access to and used their school toilet, and all households had an improved water source, a 36.05% decrease in *Ascaris* spp*.* infection was predicted. In the scenario where all participants cleaned themselves with water after defecating and all participants had wealth equivalent to those in SES quintile 3, 4, or 5, an 11.70% decrease in hookworm infection was predicted.

**Table 4:** Scenarios from tree augmented naïve (TAN) networks.

	Predicted % infection	Change in % infection
**Scenarios for *Ascaris* spp. infection**		
**A:** All schools have toilets that are used by participants	5.19	−34.01
**B:** All household water sources are tubewell or piped	31.30	−7.90
**C:** Scenario A and B combined	3.15	−36.05
**Scenarios for hookworm infection**		
**A:** All participants clean themselves with water after defecating	5.52	−9.18
**B:** All participants same wealth as participants in socioeconomic quintile 3, 4 and 5	9.29	−5.41
**C:** Scenario A and B combined	3.00	−11.70

In each scenario the response category under investigation was set to 100%. “Predicted % infection” is the predicted prevalence of *Ascaris* spp. infection in each scenario. “Change in % infection” refers to the difference between predicted prevalence in each scenario and the existing prevalence in the actual data (*Ascaris* spp. 39.2%; hookworm 14.7%).

**Supplementary figure S3:**
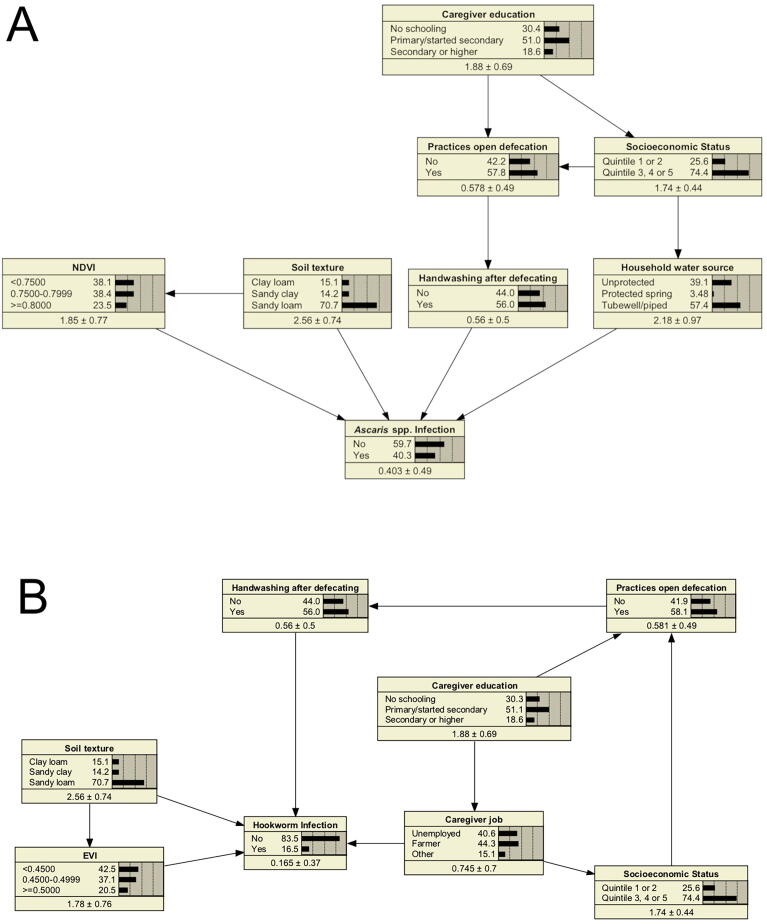
Expert structured Bayesian networks for (A) Ascaris spp. and (B) hookworm infections. Expert structured models attempt to show more complex relationships between variables based on existing literature on Ascaris spp. and hookworm transmission. Number values in each node box represent respective variable response probabilities based on 100% of data. Normalised difference vegetation index (NDVI) in (A) and enhanced vegetation index EVI in (B) refer to vegetation indexes taking values between -1 and 1 (more vegetation). Temperature and precipitation data are averaged from 1970-2000.

Results of expert structured models are shown in Supplementary Fig. S3. Scenario analyses for expert structured BNs are not presented as they were outperformed by the TAN networks.

**Fig. 4:**
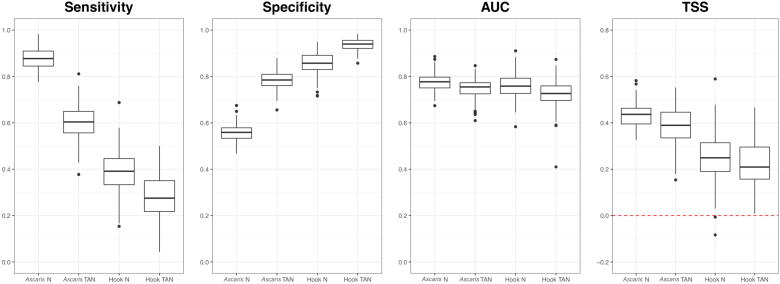
Model diagnostics for *Ascaris* spp. and hookworm naïve and tree augmented naïve (TAN) networks. Five number metrics are displayed for each model by each of the four diagnostic types: sensitivity, specificity, area under the curve (AUC) and true skill statistic (TSS). *Ascaris* N, *Ascaris* spp. naïve model; *Ascaris* TAN, *Ascaris* spp. TAN model; Hook N, hookworm naïve model; Hook TAN, Hookworm TAN model. Sensitivity, specificity and AUC are presented as a decimal out of 1. TSS can have values between −1 and 1, where positive values indicate better performance than random classification.

Model diagnostics indicated little overall variation in median AUC values across the 100 trials, but a greater range of AUC values between trials was observed for the hookworm TAN model (Fig. 4). Only positive values (excluding outliers) for TSS were observed in all models. Hookworm naïve and TAN models had lower median TSSs compared with *Ascaris* spp. models. Overall, hookworm models tended to have higher specificity compared with both *Ascaris* spp. models, and the opposite was observed for sensitivity.

### 3.5 Comparisons among techniques

Table 5 provides a summary of risk factors identified by each modelling approach. For *Ascaris* spp., only NDVI was identified across all three statistical techniques. For hookworm, cleaning oneself with water after defecating was identified across all techniques and only BNs identified environmental factors. RP classification trees identified the most demographic and WASH variables. Logistic regression identified the fewest variables overall, with no variables uniquely identified for hookworm.

**Table 5:** Summary of risk factors identified by logistic regression (LR), recursive partitioning (RP) and Bayesian networks (BN) techniques for *Ascaris* spp. and hookworm outcomes.

Predictor	Techniques that identified predictor as a risk factor	
	*Ascaris* spp.	Hookworm
School toilet use	LR, BN	
Household water treated by boiling	LR	
Household toilet has slab	RP	
Household water availability	RP	
Washes hands after defecating		RP
Cleans themself with water after defecating		**LR, RP, BN**
Shoe wearing outside home	RP	
Age	RP	LR, RP
More than six people in household		RP
Socioeconomic status		LR, BN
Elevation	RP, BN	
Household water source	BN	
Precipitation^a^	BN	BN
Slope	BN	
Soil texture	BN	
Soil pH	BN	
Land cover	BN	
EVI		
Temperature^b^	LR, BN	BN
NDVI	**LR, RP, BN**	

EVI, enhanced vegetation index; NDVI, normalised difference vegetation index.

Risk factors presented for BNs refer to the naïve models which were the same for tree augmented naïve (TAN) models.

a Different variations of the precipitation variable were identified by *Ascaris* spp. BN (mean precipitation in wettest quarter, monthly average precipitation) and for hookworm BN (mean precipitation in driest month).

b Different variations of the temperature variable were identified by BN (mean temperature in warmest quarter, annual mean temperature) and LR (mean temperature in coldest month).

## 4 Discussion

Studies examining WASH and environmental risk factors for STH infections have not consistently identified the same variables associated with infection. Almost all studies have used the standard LR approach. In this study we hypothesised that using alternative statistical techniques may provide further insights and more robust analyses for identifying risk factors. In analysing the same data by LR and the more novel techniques of RP and BNs, our study provided additional insights into risk factors for STH infections.

For *Ascaris* spp., NDVI (vegetation index) was the only variable consistently identified across all techniques. A previous study from Timor-Leste using LR also found NDVI to be associated with *Ascaris* spp*.* infection (Campbell et al., 2017). Higher NDVI suggests denser vegetation, indicative of tropical regions with moist soil that are ideal for survival of STH eggs and larvae (Gordon et al., 2017). For hookworm, only cleaning oneself with water after defecating was consistently identified by all techniques.

For both infection outcomes, fewer influential variables were identified by LR. This may be due to the limitation of LR when handling correlated data (Spratt et al., 2013, Ranganathan et al., 2017). In RP, the model considers which variable is most strongly associated with the outcome, conditional on all the variables selected prior (Gass et al., 2014). While some issues with accuracy of measurement error have been raised in other contexts (Zeger et al., 2000, Tolbert et al., 2007, Strobl et al., 2009), this approach enables all correlated variables in the dataset to be considered. Notably, of the three techniques, RP identified the largest number of WASH variables. BNs can also model correlated variables as seen in our naïve and TAN models that included many environmental variables, whereas in LR, most were removed due to collinearity.

In terms of model performance, median AUC and TSS were similar for all three types of models. Specificity was higher than sensitivity across all models, consistent with a RP analysis of WASH risk factors for STH infections, likely due to the imbalanced data sample having more non-infected than infected participants (Gass et al., 2014). This may also explain why hookworm models tended to have lower sensitivity compared with the respective *Ascaris* spp. models as there was a higher prevalence of *Ascaris* spp. infection.

One of the major benefits of LR is its use in multi-level modelling where accounting for clustering is possible. Our findings suggest school level variation was important in our final multivariable LR models. One previous study has demonstrated a method of accounting for clustering in BNs; however, this was limited to naïve networks (Fernández et al., 2014). There are no known methods of accounting for clustering in RP (Gass et al., 2014). To attempt to address this limitation, the school variable was included as a fixed effect in BN and RP models. For RP, the school variable was the first partition in both classification trees, indicating that all other variables identified were conditional on school. It is possible then that some variables may not have been identified if they did not have a conditional relationship with school. In BNs, both TAN models revealed that almost all variables had a relationship with the school variable. This leads to limitations in drawing causal inferences from our BNs which is often an attractive reason for using this method (Joffe et al., 2012).

Both BNs and RP produced graphical representations that provided novel insights beyond identifying risk factors. In RP, the *Ascaris* spp. and hookworm classification trees depicted eight and six outcome groups respectively. For some outcomes, much higher prevalence of infection was predicted, which may indicate at-risk population sub-groups (Lemon et al., 2003). BNs have the advantage of visualising relationships between variables, and being able to model scenarios in real-time without further analyses (Lau et al., 2017). While odds ratios have the benefit of having effect sizes for predictors which are critical in research processes such as determining drug efficacy (Knol et al., 2012), the novel insights from RP and BNs provide a more informative and holistic evidence base for informing policy.

This study had several important limitations. Given the small sample size, risk factors are not generalisable. Additionally, LR and RP diagnostics were derived from fewer trials than BNs and should therefore be compared cautiously. Directions for future studies include using a larger dataset, increasing trials for model diagnostics and exploring other types of BNs and ensembles of trees produced from RP. A larger sample would enable school-level analysis to overcome the inability to account for clustering in RP and BNs, and may also improve the performance of expert structured BNs by providing more examples to train models.

To the best of our knowledge, this is the first study that used the same dataset to compare LR, BNs and RP in the context of risk factor identification for STH infections. While BNs and RP are used in other areas of public health (Martha Maria et al., 2017, Arora et al., 2019, Wang et al., 2020), such techniques have rarely been utilised in NTD research. An additional strength of this study is the inclusion of a wide of range of variables including environmental, demographic, and WASH access and behaviours. This revealed trends in the risk factors identified by each statistical technique. While these techniques have different approaches and capacities, considerations were also made to keep variables as consistent as possible across techniques.

As research methods continue to evolve, alternative analytical approaches must be explored. As shown in our study, such approaches can reveal novel insights to support more robust evidence-based conclusions. Such evidence may contribute to the development and implementation of tailored interventions to help achieve sustainable STH control.
