# Supplementary material for: Novel statistical approaches to identify risk factors for soil-transmitted helminth infection in Timor-Leste
Source: Int J Parasitol. 2021 Aug;51(9):729–39. doi: 10.1016/j.ijpara.2021.01.005 (PMC8378505; doi:10.1016/j.ijpara.2021.01.005)
Supplement: Supplementary data 1 [file mmc1.docx]

**Supplementary Table S1.** School and community characteristics of populations participating in this study.

| **School^a^** | ***n* (%*Ascaris* spp. infection)^b^** | ***n* (% hookworm infection)^b^** | **Community**  **no.^a^** | **Participating students residing in each community  (*n*)** | **Total population of each community  (*N*)^c^** | ***n* (%*Ascaris* spp. infection)^d^** | ***n* (% hookworm infection)^d^** |
| --- | --- | --- | --- | --- | --- | --- | --- |
| **Total**  **(*n*=464)** | 182 (39.2) | 68 (14.7) |  |  |  |  |  |
| A (*n*=101) | 47 (46.5) | 34 (33.7) | 1 | 12 | 259 | 9 (75) | 3 (25) |
|  |  |  | 2 | 81 | 558 | 34 (41.9) | 27 (33.3) |
|  |  |  | 3 | 6 | 425 | 2 (33.3) | 4 (66.7) |
|  |  |  | 4 | 2 | 130 | 2 (100) | 0 |
| B (*n*=197) | 120 (60.9) | 10 (5.1) | 5 | 15 | 109 | 6 (40) | 1 (6.7) |
|  |  |  | 6 | 72 | 313 | 50 (69.4) | 4 (5.6) |
|  |  |  | 7 | 7 | 446 | 3 (42.8) | 0 |
|  |  |  | 8 | 7 | 253 | 5 (71.4) | 1 (14.3) |
|  |  |  | 9 | 77 | 344 | 48 (62.3) | 2 (2.6) |
|  |  |  | 10 | 15 | 242 | 6 (40) | 2 (13.3) |
|  |  |  | 11 | 4 | 172 | 2 (50) | 0 |
| C (*n*=28) | 3 (10.7) | 7 (25) | 12 | 28 | 364 | 3 (10.7) | 7 (25) |
| D (*n*=38) | 4 (10.5) | 6 (15.8) | 13 | 37 | 331 | 4 (12.1) | 6 (16.2) |
|  |  |  | 14 | 1 | 217 | 0 | 0 |
| E (*n*=63) | 7 (11.1) | 9 (14.3) | 15 | 63 | 1055 | 7 (11.1) | 9 (14.3) |
| F (*n*=37) | 1 (2.7) | 2 (5.6) | 16 | 1 | 643 | 0 | 0 |
|  |  |  | 17 | 36 | 614 | 1 (2.8) | 2 (5.6) |
|  |  |  |  |  |  |  |  |

*n*, observation count; %, proportion.

**^a^**For confidentiality, schools are coded as letters and communities are coded as numbers. Students recruited in each school (labelled A to F) resided in nearby communities (labelled 1 to 12).

**^b^**School proportion within whole dataset.

**^c^**Total community population was sourced from the 2015 Aldeia (community) census published by Statistics Timor-Leste, <https://www.statistics.gov.tl/category/publications/census-publications/>.

**^d^**Community proportion within the same school.

**Supplementary Table S2.** List of variables considered in this study.

|  | **Coding for LR** | **Coding for RP** | **Coding for BN** |
| --- | --- | --- | --- |
| **General variables** |  |  |  |
| Age at baseline, in years | Continuous | Continuous | Binary (1 = under 12, 2 = over 12) |
| Sex | Binary (0 = Female; 1 = Male) | Binary | Binary |
| **Individual hygiene** |  |  |  |
| Washes hands with soap or ash | Binary (1 = Yes; 0 = No) | Binary | Binary |
| Handwashing before food contact | Binary (1 = Yes; 0 = No) | Binary | Binary |
| Handwashing after fecal contact | Binary (1 = Yes; 0 = No) | Binary | Binary |
| Handwashing after contact with dirty objects | Binary (1 = Yes; 0 = No) | Binary | Binary |
| Shoe wearing in home | Binary (1 = Yes; 0 = No) | Binary | Binary |
| Shoe wearing outside home | Binary (1 = Yes; 0 = No) | Binary | Binary |
| Shoe wearing when defecating or urinating | Binary (1 = Yes; 0 = No) | Binary | Binary |
| **Individual sanitation** |  |  |  |
| Main place of defecation is toilet | Binary (1 = Yes; 0 = No) | Binary | Binary |
| Practices open defecation | Binary (1 = Yes; 0 = No) | Binary | Binary |
| Cleaning oneself with water after defecating | Binary (1 = Yes; 0 = No) | Binary | Binary |
| **School sanitation** |  |  |  |
| School toilet use | Categorical   0 = School does not have toilet  1 = Does not use school toilet  2 = Uses school toilet | Ordinal (0,1,2) | Categorical |
| **Caregiver socioeconomic variables** |  |  |  |
| Caregiver education | Categorical   1 = Never went to school  2 = Not finished primary  3 = Finished primary but not secondary  4 = Finished secondary or higher | Ordinal (1,2,3,4) | Categorical  1 = No schooling  2 = Primary/started secondary  3 = Finished Secondary or higher |
| Caregiver job type | Categorical  0 = Unemployed  1 = Farmer   2 = Other job | Ordinal (0,1,2) | Categorical |
| **Household sanitation** |  |  |  |
|  |  |  |  |
| Whether household toilet has water | N/A | Ordinal (0,1,2) | Categorical  0 = No toilet  1 = Toilet without water  2 = Toilet with water |
| Whether household toilet has slab | N/A | Ordinal (0,1,2) | Categorical  0 = No toilet  1 = Toilet without slab  2 = Toilet with slab |
| Whether household toilet has slab or water | Categorical  0 = No toilet   1 = Toilet with only water or only slab  2 = Toilet with water and slab |  |  |
| **Household water** |  |  |  |
| Household water source | Categorical  1 = Surface water  2 = Unprotected spring/dugwell  3 = Protected spring  4 = Tubewell/piped shared  5 = Piped to dwelling/yard | Ordinal (1,2,3,4,5) | Categorical  1 = Surface/unprotected   2 = Protected spring  3 = Tubewell/piped |
| Distance to water source | Binary (0 = <15 min, 1 = 15 mins or more) | Binary | Binary |
| Water source availability | Binary (1 = Water unavailable more than one week per month; 0 = Water always available or available more than three weeks per month) | Binary | Binary |
| Water always stored in covered containers | Binary (1 = Yes; 0 = No) | Binary | Binary |
| Water treated by boiling | Binary (1 = Yes; 0 = No) | Binary | Binary |
| **Household socioeconomic** |  |  |  |
| Children under 5 years in household | Binary (1 = Yes; 0 = No) | Binary | Binary |
| More than six people in household | Binary (1 = Yes; 0 = No) | Binary | Binary |
| Socioeconomic status | Categorical  2 = quintile 1 or 2  3 = quintile 3  4 = quintile 4   5 = quintile 5 | Ordinal (2,3,4,5) | Binary (1 = quintile 1 or 2,  2 = quintile 3, 4 or 5) |
| **Environmental** |  |  |  |
| Average EVI (factor of 1000) | Continuous | Continuous | Categorical (<4500, 4500-4999, 5000+) |
| Average NDVI (factor of 1000) | Continuous | Continuous | Categorical (<7500, 7500-7999, 8000+) |
| Land cover | Categorical  1 = Croplands  2 = Grasslands  3 = Savanna  4 = Woody savanna | Categorical | Categorical  1 = Croplands  2 = Grasslands  3 = Savanna/woody savanna |
| Slope | Continuous in ° | Continuous in ° | Categorical (<10, 10-19.99, 20+) |
| Elevation | Continuous in m | Continuous in m | Categorical (<500, 500-999, 1000+) |
| Monthly average precipitation | Continuous in mm | Continuous in mm | Categorical (<150, 150-174.99, 175+) |
| Precipitation in driest quarter (August - October) | Continuous in mm | Continuous in mm | Categorical (<25, 25-29.99, 30+) |
| Precipitation in wettest quarter (December - February) | Continuous in mm | Continuous in mm | Categorical (<300, 300-349.9, 350+) |
| Precipitation in driest month (September) | Continuous in mm | Continuous in mm | Categorical (<15, 15-15.99, 16+) |
| Precipitation in wettest month (February) | Continuous in mm | Continuous in mm | Categorical (<300, 300-349.99, 350+) |
| Annual Mean temperature | Continuous in ° C | Continuous in ° C | Categorical (<20, 20-24.99, 25+) |
| Temperature in warmest quarter (October - December) | Continuous in ° C | Continuous in ° C | Categorical (<22, 22-23.99, 24+) |
| Temperature in coldest quarter (June - August) | Continuous in ° C | Continuous in ° C | Categorical (<20, 20-23.99, 24+) |
| Temperature in hottest month (November) | Continuous in ° C | Continuous in ° C | Categorical (<27.5, 27.5-29.99, 30+) |
| Temperature in coldest month (July) | Continuous in ° C | Continuous in ° C | Categorical (<12.5, 12.5-14.99, 15+) |
| Temperature range (hottest minus coldest month) | Continuous in ° C | Continuous in ° C | Categorical (<12, 12-13.9, 14+) |
| Annual maximum temperature | Continuous in ° C | Continuous in ° C | Categorical (<25, 25-29.9, 30+) |
| Annual minimum temperature | Continuous in ° C | Continuous in ° C | Categorical (<15, 15-19.9, 20+) |
| Soil texture | Categorical   1 = Clay loam  2 = Sandy clay  3 = Sandy loam | Ordinal (1,2,3) | Categorical |
| Soil pH (3 categories) | Categorical  1 = Acidic (5.5-6.49)  2 = Neutral (6.5-7.29)  3 = Basic (7.3-8.4) | Ordinal (1,2,3) | Categorical |
| Soil pH (5 categories) | Categorical  1 = Moderately acidic (5.5-5.99)  2 = Slightly acidic (6.0-6.49  3 = Neutral (6.5-7.29)  4 = Slightly alkaline (7.3-7.79)  5 = Moderately alkaline (7.8-8.4) | Ordinal (1,2,3,4) | N/A |

LR, logistic regression; RP, recursive partitioning; BN, Bayesian networks; EVI, enhanced vegetation index; NDVI, normalised difference vegetation index.

**Supplementary Table S3.** Results of descriptive analysis for demographic and water, sanitation and hygiene variables.

|  | **LR and RP categorisations** | | | **BN categorisations** | | | |
| --- | --- | --- | --- | --- | --- | --- | --- |
| **Demographics** | ***n*** | **%** | **95% CI** | **Categories** | ***n*** | **%** | **95% CI** |
| Mean age^a^ | 464 | 9.07 | 8.15—9.99 | Under 12 years | 376 | 81.03 | 68.41—89.4 |
|  |  |  |  | 12 years and over | 88 | 18.97 | 10.60—31.59 |
| Sex |  |  |  |  |  |  |  |
| Male | 229 | 49.35 | 44.29—54.43 |  |  |  |  |
| Female | 235 | 50.65 | 45.57—55.71 |  |  |  |  |
| **STH infections** |  |  |  |  |  |  |  |
| *Ascaris* spp. infection | 182 | 39.22 | 15.11—70.06 |  |  |  |  |
| Any hookworm infection | 68 | 14.66 | 0.44—39.23 |  |  |  |  |
| *Necator Americanus* infection | 64 | 13.79 | 4.50—35.19 |  |  |  |  |
| *Ancylostoma* spp. infection | 4 | 0.86 | 0.07—10.33 |  |  |  |  |
| **Individual hygiene** |  |  |  |  |  |  |  |
| Uses soap/ash when washing hands | 393 | 84.7 | 74.88—91.13 |  |  |  |  |
| Washes hands before eating and food preparation | 157 | 33.83 | 25.15—43.76 |  |  |  |  |
| Washes hands after contact with faeces | 260 | 56.03 | 39.23—71.56 |  |  |  |  |
| Washes hands after contact with dirty objects | 412 | 88.79 | 82.53—93.00 |  |  |  |  |
| Always wears shoes inside | 297 | 64.43 | 56.89—71.31 |  |  |  |  |
| Always wears shoes outside | 305 | 66.16 | 57.32—74.00 |  |  |  |  |
| Always wears shoes when defecating or urinating | 309 | 67.03 | 58.86—74.28 |  |  |  |  |
| **Individual sanitation** |  |  |  |  |  |  |  |
| Main place of defecation is toilet | 305 | 66.02 | 40.82—84.55 |  |  |  |  |
| Practices open defecation | 267 | 57.79 | 48.71—66.37 |  |  |  |  |
| Cleaning oneself with water after defecating | 222 | 48.58 | 33.52—63.90 |  |  |  |  |
| **School sanitation** |  |  |  |  |  |  |  |
| School does not have toilet | 357 | 77.44 | 36.96—95.26 |  |  |  |  |
| School has toilet - does not use | 85 | 18.44 | 2.80—63.97 |  |  |  |  |
| School has toilet - uses school toilet | 19 | 4.12 | 0.35—34.66 |  |  |  |  |
| **Caregiver socioeconomic** |  |  |  |  |  |  |  |
| Highest level of education |  |  |  |  |  |  |  |
| Never went to school | 139 | 30.22 | 23.91—37.37 | Never went to school | 139 | 30.22 | 23.91—37.37 |
| Did not finish primary school | 112 | 24.35 | 17.85—32.77 | Primary/started secondary | 235 | 51.09 | 43.93—58.20 |
| Finished primary school but not secondary school | 123 | 26.74 | 20.93—33.47 |  |  |  |  |
| Finished secondary school or higher | 86 | 18.7 | 11.67—28.59 | Finished secondary school or higher | 86 | 18.7 | 11.67—28.59 |
| Current job |  |  |  |  |  |  |  |
| Farmer | 203 | 44.13 | 28.6—60.91 |  |  |  |  |
| Other | 70 | 15.22 | 10.30—21.92 |  |  |  |  |
| Unemployed | 187 | 40.65 | 25.79—57.45 |  |  |  |  |
| **Household sanitation** |  |  |  |  |  |  |  |
| Household toilet type |  |  |  |  |  |  |  |
| None | 150 | 32.4 | 14.64—57.24 |  |  |  |  |
| Pit latrine | 308 | 66.52 | 40.40—85.35 |  |  |  |  |
| Composting toilet | 5 | 1.08 | 0.13—8.59 |  |  |  |  |
| Household toilet has slab | 214 | 47.87 | 33.33—62.78 |  |  |  |  |
| Household toilet has water | 209 | 45.63 | 33.09—58.75 |  |  |  |  |
| **Household water** |  |  |  |  |  |  |  |
| Main household water source |  |  |  |  |  |  |  |
| Surface water | 83 | 18.04 | 7.38—37.81 | Surface water/unprotected spring/  dugwell | 180 | 39.13 | 22.10—59.29 |
| Unprotected spring/dugwell | 97 | 21.09 | 13.38—31.62 |  |  |  |  |
| Protected spring | 16 | 3.48 | 0.70—15.57 | Protected spring | 16 | 3.48 | 0.70—15.57 |
| Piped shared/tubewell | 249 | 54.13 | 35.50—71.45 | All piped/tubewell | 264 | 57.39 | 38.51—74.34 |
| Piped to dwelling | 15 | 3.26 | 1.91—5.50 |  |  |  |  |
| Distance to main household water source |  |  |  |  |  |  |  |
| Less than 15 mins | 363 | 78.23 | 64.48—87.68 |  |  |  |  |
| More than 15 mins | 101 | 21.77 | 12.32—35.52 |  |  |  |  |
| Main household water source is always available | 375 | 81.17 | 69.37—89.13 |  |  |  |  |
| All water stored in covered containers | 216 | 51.18 | 42.89—59.42 |  |  |  |  |
| Household water is treated by boiling | 385 | 83.88 | 46.35—96.91 |  |  |  |  |
| **Household socioeconomic** |  |  |  |  |  |  |  |
| More than 6 people in household | 362 | 78.02 | 51.75 — 92.15 |  |  |  |  |
| Children under 5 years in household | 205 | 44.18 | 35.93 — 52.76 |  |  |  |  |
| Socioeconomic Quintile |  |  |  |  |  |  |  |
| 1 (poorest) | 7 | 1.51 | 0.53—4.16 | Quintile 1 or 2 | 119 | 25.65 | 17.78—35.50 |
| 2 | 112 | 24.14 | 16.13—34.48 |  |  |  |  |
| 3 | 105 | 22.63 | 16.73—29.86 | Quintile 3, 4 or 5 | 345 | 3.47 | 64.50—82.23 |
| 4 | 114 | 24.57 | 16.95—34.20 |  |  |  |  |
| 5 (wealthiest) | 126 | 27.16 | 14.45—45.14 |  |  |  |  |

*n*, observation count; %, proportion; 95% CI, 95% confidence interval; LR, logistic regression; RP, recursive partitioning; BN, Bayesian networks; STH, soil-transmitted helminths. LR and RP categorisations refer to the variable groupings used in LR and RP analyses. BN categorisation refers to the groupings used for Bayesian network analyses. Proportions and 95% confidence intervals were calculated accounting for school level clustering.

^a^Age in LR and RP categorisations was encoded continuously and the value denoted under “%” refers to the mean age.

**Supplementary Table S4.** Results of descriptive analysis for community level environmental variables.

|  | **LR and RP categorisations** | | | | | **BN categorisations** | | | |
| --- | --- | --- | --- | --- | --- | --- | --- | --- | --- |
| **Environmental variables** | ***n*** | **%/μ** | **95% CI** | **min** | **max** | **Categories** | ***n*** | **%** | **95% CI** |
| Mean EVI | 464 | 0.46 | 0.41—0.50 | 0.39 | 0.58 | <0.45 | 197 | 42.46 | 15.47—74.85 |
|  |  |  |  |  |  | 0.45 - 0.49 | 172 | 37.07 | 11.08—73.57 |
|  |  |  |  |  |  | >=0.5 | 95 | 20.47 | 1.87—77.64 |
| Mean NDVI | 464 | 0.75 | 0.71—0.79 | 0.68 | 0.82 | <0.75 | 177 | 38.15 | 73.56—82.73 |
|  |  |  |  |  |  | 0.75-0.79 | 178 | 38.36 | 3.92—90.46 |
|  |  |  |  |  |  | >=0.8 | 109 | 23.49 | 2.86—76.19 |
| Landcover |  |  |  | N/A | |  |  |  |  |
| Croplands | 28 | 6.03 | 0.26—61.23 |  |  | Croplands | 28 | 6.03 | 0.26—61.23 |
| Grasslands | 43 | 8.6 | 0.73—58.64 |  |  | Grasslands | 43 | 9.27 | 0.73—58.64 |
| Savanna | 329 | 70.91 | 18.47—96.33 |  |  | Savanna/woody Savanna | 393 | 84.7 |  |
| Woody Savanna | 64 | 13.79 | 0.64—79.76 |  |  |  |  |  | 36.00—98.20 |
| Mean slope (°) | 464 | 15.19 | 9.47—20.91 | 3.93 | 25.20- | <10° | 63 | 13.58 | 6.09—80.11 |
|  |  |  |  |  |  | 10- 19.9° | 308 | 66.38 | 31.58—89.41 |
|  |  |  |  |  |  | >=20° | 93 | 20.04 | 4.24—58.66 |
| Mean elevation (m) | 464 | 949.6 | 450.0—1489 | 20.8 | 1515 | <500m | 91 | 19.61 | 1.55—79.06 |
|  |  |  |  |  |  | 500-999.9m | 67 | 14.44 | 2.73—50.4 |
|  |  |  |  |  |  | >=1000m | 306 | 65.95 | 21.11—93.34 |
| Mean average monthly precipitation (mm) | 464 | 164.8 | 140.0—189.6 | 103 | 180 | <150mm | 91 | 19.61 | 1.56—79.06 |
|  |  |  |  |  |  | 150-174.9mm | 68 | 14.66 | 2.71—51.4 |
|  |  |  |  |  |  | >=175mm | 305 | 65.73 | 20.81—93.33 |
| Mean precipitation in driest quarter (mm) | 464 | 26.88 | 21.58—32.17 | 15.5 | 34.3 | <25mm | 91 | 19.61 | 1.56—79.06 |
|  |  |  |  |  |  | 25-29.9mm | 298 | 64.22 | 1.22—95.87 |
|  |  |  |  |  |  | >=30mm | 75 | 16.16 | 1.4—72.28 |
| Mean precipitation in wettest quarter (mm) | 464 | 321.1 | 258.3—383.9 | 191 | 366 | <300mm | 91 | 19.61 | 1.56—79.06 |
|  |  |  |  |  |  | 300-349.9mm | 158 | 34.05 | 7.62—76.37 |
|  |  |  |  |  |  | >=350mm | 215 | 46.33 | 9.72—87.38 |
| Mean precipitation in driest month (mm) | 464 | 14.9 | 12.81—16.99 | 10 | 17.3 | <15mm | 63 | 13.58 | 0.61—80.11 |
|  |  |  |  |  |  | 15-15.9mm | 227 | 48.92 | 5.03—94.54 |
|  |  |  |  |  |  | >=16mm | 174 | 37.5 | 3.83—90.03 |
| Mean precipitation in wettest month (mm) | 464 | 332.4 | 260.5—404.9 | 188 | 348 | <300mm | 91 | 19.61 | 1.56—79.06 |
|  |  |  |  |  |  | 300-349.9mm | 30 | 6.47 | 0.99—32.4 |
|  |  |  |  |  |  | >=350mm | 343 | 73.92 | 23.21—95.34 |
| Mean annual mean temperature (° C) | 464 | 21.36 | 18.61—24.11 | 18.3 | 26.3 | <20°C | 203 | 43.75 | 7.39—88.35 |
|  |  |  |  |  |  | 20- 24.9°C | 170 | 36.64 | 7.09—81.42 |
|  |  |  |  |  |  | >=25°C | 91 | 19.61 | 1.55—79.06 |
| Mean temperature in warmest quarter (° C) | 464 | 22.53 | 19.89—25.16 | 19.4 | 27.3 | <22°C | 305 | 65.73 | 20.81—93.33 |
|  |  |  |  |  |  | 22- 23.9°C | 68 | 14.66 | 2.71—51.40 |
|  |  |  |  |  |  | >=24°C | 91 | 19.61 | 1.55—79.06 |
| Mean temperature in coldest quarter (° C) | 464 | 19.67 | 16.75—22.59 | 16.7 | 24.9 | <20°C | 306 | 65.95 | 21.11—93.34 |
|  |  |  |  |  |  | 20- 23.9°C | 67 | 14.44 | 2.73—50.4 |
|  |  |  |  |  |  | >=24°C | 91 | 19.61 | 1.55—79.06 |
| Mean temperature in hottest month (° C) | 464 | 27.76 | 25.42—30.13 | 24.5 | 31.9 | <27.5°C | 225 | 48.49 | 10.00—88.86 |
|  |  |  |  |  |  | 27.5- 29.9°C | 148 | 31.9 | 6.45—76.07 |
|  |  |  |  |  |  | >=30°C | 91 | 19.61 | 1.55—79.06 |
| Mean temperature in coldest month (° C) | 464 | 14.6 | 11.28—17.92 | 11.6 | 20.5 | <12.5°C | 126 | 27.16 | 7.41—63.44 |
|  |  |  |  |  |  | 12.5- 14.9°C | 180 | 15.45 | 10.63—77.15 |
|  |  |  |  |  |  | >=15°C | 158 | 34.05 | 6.66—78.89 |
| Mean temperature range (° C) | 464 | 13.16 | 11.94—14.38 | 10.9 | 14.6 | <12°C | 91 | 19.61 | 1.55—79.06 |
|  |  |  |  |  |  | 12- 13.9°C | 265 | 57.11 | 8.64—94.94 |
|  |  |  |  |  |  | >=14°C | 108 | 23.28 | 1.45—86.20 |
| Mean annual maximum temperature (° C) | 464 | 26.21 | 23.79—28.64 | 23.3 | 30.4 | <25°C | 203 | 43.75 | 7.39—88.35 |
|  |  |  |  |  |  | 25- 29.9°C | 170 | 36.63 | 7.09—81.42 |
|  |  |  |  |  |  | >=30°C | 91 | 19.61 | 1.55—79.06 |
| Mean annual minimum temperature (° C) | 464 | 16.42 | 13.33—19.50 | 13.2 | 22.1 | <15°C | 212 | 45.69 | 7.56—89.64 |
|  |  |  |  |  |  | 15- 19.9°C | 161 | 34.7 | 6.07—81.36 |
|  |  |  |  |  |  | >=20°C | 91 | 19.61 | 1.55—79.06 |
| Soil Texture |  |  |  | N/A | |  | | | |
| Clay Loam | 70 | 15.08 | 0.99—75.96 |  |  |  |  |  |  |
| Sandy Clay | 66 | 14.22 | 1.21—69.13 |  |  |  |  |  |  |
| Sandy Loam | 328 | 70.69 | 18.08—96.34 |  |  |  |  |  |  |
| Soil pH in 3 categories |  |  |  | N/A | |  | | | |
| Acidic (5.5-6.49) | 363 | 78.23 | 33.9—96.18 |  |  |  |  |  |  |
| Neutral (6.5-7.29) | 38 | 8.19 | 3.09—19.94 |  |  |  |  |  |  |
| Basic (7.3-8.4) | 63 | 13.58 | 0.61—80.11 |  |  |  |  |  |  |
| Soil pH in 5 categories |  |  |  | N/A | | Not included in BN analysis | | | |
| Moderately acidic | 148 | 31.9 | 3.77—84.84 |  |  |  |  |  |  |
| Slightly acidic | 38 | 3.19 | 3.09—19.95 |  |  |  |  |  |  |
| Neutral | 215 | 46.34 | 7.37—90.36 |  |  |  |  |  |  |
| Slightly alkaline | 63 | 13.58 | 0.61—80.11 |  |  |  |  |  |  |
| Moderately alkaline | 0 |  |  |  |  |  |  |  |  |

*n*, observation count; %, proportion; μ, mean; 95% CI, 95% confidence interval; LR, logistic regression; RP, recursive partitioning; BN, Bayesian networks; EVI, enhanced vegetation index; NDVI, normalised difference vegetation index.

NDVI and EVI are indices quantifying vegetation and are measured between -1 to 1 with values closer to 1 indicating more vegetation.

LR and RP *categorisations refers to the variable groupings used in* LR and RP *analyses.* BN *categorisations refers to the groupings used for Bayesian network analyses. Proportions and 95% CIs were calculated, adjusting for school level clustering. Variables named with “mean” were encoded continuously for LR and RP categorisations with the value denoted under “%/μ” referring to the mean. All variables in BN categorisations were categorical.*

**Supplementary Table S5.** Results of univariable and within-domain logistic regression analysis for Ascaris spp. infection.

| ***Odds of Ascaris* spp. *infection*** | **Univariable** | | | | **Within-domain** | | | |
| --- | --- | --- | --- | --- | --- | --- | --- | --- |
| **Demographic variables** | **OR** | **95% CI** | ***P*** | **No. obvs** | **aOR** | **95% CI** | ***P*** | **No. obvs** |
| Age in years^a^ | 1.02 | 0.93—1.10 | 0.695 | 464 | 1.02 | 0.94—1.11 | 0.665 | 464 |
| Male sex^b^ | 0.91 | 0.59—1.41 | 0.681 | 464 | 0.91 | 0.59—1.40 | 0.653 |  |
| **Individual hygiene variables** |  |  |  |  |  | | | |
| Handwashing with soap or ash | 0.73 | 0.39—1.34 | 0.307 | 464 |  |  |  |  |
| Washing hands before eating and preparing food | 1.26 | 0.79—1.99 | 0.330 | 464 |  |  |  |  |
| Washing hands after toileting | 0.60 | 0.38—0.94 | **0.028** | 464 | 0.57 | 0.36—0.91 | **0.019** | 464 |
| Washing hands after contact with dirt | 1.10 | 0.53—2.29 | 0.791 | 464 |  |  |  |  |
| Wearing shoes inside home | 1.24 | 0.78—1.96 | 0.357 | 461 |  |  |  |  |
| Wearing shoes outside home | 0.94 | 0.59—1.49 | 0.779 | 461 |  |  |  |  |
| Wearing shoes when defecating | 0.90 | 0.56—1.43 | 0.651 | 461 |  |  |  |  |
| **Individual sanitation variables** |  |  |  |  |  | | | |
| Main place of defecation is toilet | 1.09 | 0.67—1.78 | 0.717 | 462 |  |  |  |  |
| Practices open defecation | 0.76 | 0.49—1.17 | 0.214 | 462 |  |  |  |  |
| Cleaning oneself with water after defecating | 0.80 | 0.51—1.26 | 0.355 | 457 |  |  |  |  |
| **School sanitation**^c^ |  |  |  | 461 |  |  |  | 461 |
| Does not use school toilet | 2.09 | 1.11—3.92 | **0.022** |  | 2.15 | 1.14—4.05 | **0.019** |  |
| Uses school toilet | 0.27 | 0.28—2.57 | 0.254 |  | 0.27 | 0.03—2.60 | 0.255 |  |
| **Caregiver socioeconomic variables** |  |  |  |  |  | | | |
| Education level^d^ |  |  |  | 460 |  |  |  | 460 |
| Not finished primary | 0.58 | 0.31—1.08 | **0.084** |  | 0.62 | 0.33—1.16 | 0.134 |  |
| Finished primary not secondary | 0.88 | 0.04—1.58 | 0.670 |  | 0.97 | 0.52—1.79 | 0.911 |  |
| Finished secondary or higher | 0.51 | 0.27—0.96 | **0.038** |  | 0.58 | 0.25—1.32 | 0.191 |  |
| Caregiver's job^e^ |  |  |  | 460 |  |  |  |  |
| Unemployed | 0.70 | 0.42—1.15 | **0.159** |  | 0.77 | 0.45—1.33 | 0.352 |  |
| Other | 0.62 | 0.33—1.87 | **0.151** |  | 0.85 | 0.37—1.98 | 0.712 |  |
| **Household sanitation variables^f^** |  |  |  | 447 |  | | | |
| Toilet only and/or only slab/water | 1.35 | 0.72—2.54 | 0.353 |  |  |  |  |  |
| Toilet with slab and water | 0.86 | 0.50—1.47 | 0.579 |  |  |  |  |  |
| **Household water variables** |  |  |  |  |  | | | |
| Household water source^g^ |  |  |  | 460 |  |  |  |  |
| Unprotected spring/dugwell | 1.43 | 0.73—2.79 | 0.296 |  |  |  |  |  |
| Protected spring | 1.88 | 0.44—8.05 | 0.396 |  |  |  |  |  |
| Tubewell/piped shared | 0.82 | 0.45—1.51 | 0.528 |  |  |  |  |  |
| Piped to dwelling | 0.52 | 0.13—2.03 | 0.349 |  |  |  |  |  |
| Water source distance more than 15 min^h^ | 0.84 | 0.51—1.39 | 0.497 | 464 |  |  |  |  |
| Water unavailable >1 week per month^i^ | 1.03 | 0.59—1.78 | 0.922 | 462 |  |  |  |  |
| Water always covered | 0.87 | 0.55—1.36 | 0.531 | 422 |  |  |  |  |
| Water treated by boiling | 0.30 | 0.13—0.68 | **0.004** | 459 | 0.30 | 0.13—0.68 | **0.004** | 459 |
| **Household socioeconomic variables** |  |  |  |  |  | | | |
| Has children under 5 in household | 0.78 | 0.49—1.21 | 0.267 | 464 |  |  |  |  |
| More than 6 people in household | 0.93 | 0.51—1.69 | 0.816 | 464 |  |  |  |  |
| Socioeconomic quintile^j^ |  |  |  | 464 |  |  |  |  |
| 3 | 1.34 | 0.70—2.57 | 0.384 |  | 1.34 | 0.70—2.57 | 0.381 | 464 |
| 4 | 0.68 | 0.36—1.28 | 0.231 |  | 0.68 | 0.36—1.28 | 0.231 |  |
| 5 | 0.53 | 0.28—1.00 | **0.050** |  | 0.53 | 0.28—1.01 | **0.053** |  |
| **Community-level environmental variables** |  |  |  |  |  | | | |
| Annual mean temperature^a^ | 0.74 | 0.55—0.99 | **0.042** | 464 |  |  |  |  |
| Annual max temperature^a^ | 0.72 | 0.53—0.97 | **0.032** | 464 |  |  |  |  |
| Annual min temperature^a^ | 0.76 | 0.57—1.01 | **0.056** | 464 |  |  |  |  |
| Mean temperature warmest quarter^a^ | 0.74 | 0.55—0.99 | **0.046** | 464 |  |  |  |  |
| Mean temperature coldest quarter^a^ | 0.75 | 0.60—0.94 | **0.014** | 464 |  |  |  |  |
| Temperature in hottest month^a^ | 0.73 | 0.55—0.98 | **0.037** | 464 |  |  |  |  |
| Temperature in coldest month^a^ | 0.77 | 0.62—0.95 | **0.013** | 464 | 0.72 | 0.56—0.92 | **0.009** |  |
| Temperature range^a^ | 2.14 | 1.17—3.92 | **0.013** | 464 |  |  |  |  |
| Slope^a^ | 1.08 | 0.99—1.19 | **0.086** | 464 | 1.03 | 0.92—1.15 | 0.569 |  |
| Elevation^a^ | 1.00 | 1.00—1.00 | **0.020** | 464 |  |  |  |  |
| EVI^a^ (factor of 100) | 1.00 | 0.93—1.08 | 0.988 | 464 |  |  |  |  |
| NDVI^a^ (factor of 100) | 1.10 | 1.01—1.20 | **0.025** | 464 | 1.13 | 0.99—1.29 | **0.076** |  |
| Monthly average precipitation^a^ | 1.02 | 0.99—1.06 | **0.191** | 464 |  |  |  |  |
| Mean precipitation in driest quarter^a^ | 1.02 | 0.85—1.21 | 0.857 | 464 |  |  |  |  |
| Mean precipitation in wettest quarter^a^ | 1.01 | 0.99—1.02 | 0.219 | 464 |  |  |  |  |
| Precipitation in driest month^a^ | 0.88 | 0.51—1.51 | 0.635 | 464 |  |  |  |  |
| Precipitation in wettest month^a^ | 1.01 | 1.00—1.02 | **0.194** | 464 |  |  |  |  |
| Land cover^k^ |  |  |  | 464 |  |  |  |  |
| Grassland | 1.17 | 0.07—18.46 | 0.910 |  |  |  |  |  |
| Savanna | 4.51 | 0.37—54.97 | 0.237 |  |  |  |  |  |
| Woody Savanna | 1.04 | 0.05—20.66 | 0.980 |  |  |  |  |  |
| Soil texture^l^ |  |  |  | 464 |  |  |  |  |
| Sandy Loam | 0.28 | 0.02—3.75 | 0.336 |  |  |  |  |  |
| Sandy Clay | 0.84 | 0.15—4.58 | 0.836 |  |  |  |  |  |
| Soil pH in 3 categories^m^ |  |  |  | 464 |  |  |  |  |
| neutral | 1.39 | 0.50—3.86 | 0.525 |  |  |  |  |  |
| basic | 0.46 | 0.03—8.39 | 0.602 |  |  |  |  |  |
| Soil pH in 5 categories^n^ |  |  |  | 464 |  |  |  |  |
| slightly acidic | 2.69 | 0.97—7.50 | **0.058** |  | 0.55 | 0.14—2.17 | 0.393 |  |
| neutral | 2.22 | 0.82—6.07 | **0.118** |  | 0.59 | 0.15—2.41 | 0.466 |  |
| slightly alkaline | 0.72 | 0.04—12.43 | 0.821 |  | 1.22 | 0.05—31.51 | 0.906 |  |

OR, odds ratio; aOR, odds ratio adjusting for age and sex; 95% CI, 95% confidence interval; No. of obvs., number of observations; EVI, enhanced vegetation index; NDVI, normalised difference vegetation index.

NDVI and EVI are indices quantifying vegetation and are measured between -1 to 1 with values closer to 1 indicating more vegetation. OR and aOR for NDVI refer to an 0.01 unit increase in NDVI as the variable was transformed by a factor of 100.

^a^Variable was encoded continuously. Reference categories are as follows: ^b^female; ^c^school has no toilet; ^d^no schooling; ^e^farmer; ^f^no toilet; ^g^surface water; ^h^water source less than 15 min; ^i^water always available or unavailable < 1 week/month; ^j^quintile 1 or 2; ^k^croplands, ^l^clay loam, ^m^acidic, ^n^moderately acidic.

**Supplementary Table S6.** Results of univariable and within-domain logistic regression analysis for hookworm infection.

| ***Odds of any hookworm infection*** | **Univariable** | | | | **Within-domain** | | | |
| --- | --- | --- | --- | --- | --- | --- | --- | --- |
| **Demographic variables** | **OR** | **95% CI** | ***P*** | **No. obvs** | **aOR** | **95% CI** | ***P*** | **No. obvs** |
| Age in years^a^ | 1.26 | 1.13—1.42 | **<0.001** | 464 | 1.26 | 1.12—1.41 | **<0.001** | 464 |
| Male sex^b^ | 1.30 | 0.76—2.25 | 0.339 | 464 | 1.19 | 0.68—2.09 | 0.541 |  |
| **Individual hygiene variables** |  |  |  |  |  | | | |
| Handwashing with soap or ash | 1.78 | 0.77—4.10 | **0.176** | 464 | 1.85 | 0.77—4.47 | 0.171 | 461 |
| Washing hands before eating and preparing food | 1.12 | 0.63—1.98 | 0.704 | 464 |  |  |  |  |
| Washing hands after toileting | 1.00 | 0.57—1.76 | 0.989 | 464 |  |  |  |  |
| Washing hands after contact with dirt | 1.29 | 0.51—3.28 | 0.596 | 464 |  |  |  |  |
| Wearing shoes inside home | 1.54 | 0.84—2.82 | **0.161** | 461 | 1.26 | 0.67—2.40 | 0.472 |  |
| Wearing shoes outside home | 1.22 | 0.67—2.23 | 0.510 | 461 |  |  |  |  |
| Wearing shoes when defecating | 1.45 | 0.78—2.69 | 0.234 | 461 |  |  |  |  |
| **Individual sanitation variables** |  |  |  |  |  | | | |
| Main place of defecation is toilet | 0.67 | 0.38—1.18 | **0.165** | 462 | 2.19 | 0.91—5.23 | **0.079** | 457 |
| Practices open defecation | 1.84 | 1.02—3.31 | **0.043** | 462 | 1.35 | 0.57—3.21 | 0.493 |  |
| Cleans oneself with water after defecating | 0.29 | 0.15—0.54 | **<0.001** | 457 | 0.23 | 0.10—0.52 | **<0.001** |  |
| **School sanitation**^c^ |  |  |  | 461 |  | | | |
| Does not use school toilet | 0.89 | 0.28—2.85 | 0.844 |  |  |  |  |  |
| Uses school toilet | 0.51 | 0.05—5.02 | 0.563 |  |  |  |  |  |
| **Caregiver socioeconomic variables** |  |  |  |  |  | | | |
| Education level^d^ |  |  |  | 460 |  |  |  | 460 |
| Not finished primary | 0.85 | 0.43—1.71 | 0.652 | 460 | 0.75 | 0.37—1.55 | 0.443 | 460 |
| Finished primary not secondary | 0.63 | 0.31—1.30 | 0.216 |  | 0.67 | 0.31—1.45 | 0.308 |  |
| Finished secondary or higher | 0.29 | 0.11—0.78 | **0.014** |  | 0.29 | 0.08—1.01 | **0.052** |  |
| Caregiver's job^e^ |  |  |  | 460 |  |  |  |  |
| Unemployed | 0.66 | 0.35—1.24 | **0.193** |  | 0.87 | 0.44—1.74 | 0.669 |  |
| Other | 0.59 | 0.25—1.40 | 0.234 |  | 1.41 | 0.46—4.35 | 0.547 |  |
| **Household sanitation variables**^f^ |  |  |  |  |  | | | |
| Toilet only and/or only slab/water | 1.29 | 0.61—2.71 | 0.509 | 447 | 1.20 | 0.57—2.52 | 0.631 | 447 |
| Toilet with slab and water | 0.50 | 0.26—0.97 | **0.039** |  | 0.54 | 0.27—1.08 | **0.083** |  |
| **Household water variables** |  |  |  |  |  | | | |
| Household water source^g^ |  |  |  | 460 |  |  |  | 455 |
| Unprotected spring/dugwell | 1.98 | 0.77—5.08 | **0.154** |  | 2.84 | 1.03—7.83 | **0.043** |  |
| Protected spring | 2.53 | 0.55—11.56 | **0.231** |  | 4.40 | 0.84—23.18 | **0.080** |  |
| Tubewell/piped shared | 1.11 | 0.46—2.67 | 0.813 |  | 1.64 | 0.62—4.29 | 0.316 |  |
| Piped to dwelling | 2.41 | 0.56—10.27 | 0.236 |  | 4.88 | 1.03—23.23 | **0.046** |  |
| Water source distance more than 15 min^h^ | 1.55 | 0.83—2.90 | **0.168** | 464 | 1.62 | 0.79—3.31 | 0.188 |  |
| Water unavailable >1 week per month^i^ | 1.38 | 0.70—2.71 | 0.349 | 462 |  |  |  |  |
| Water always covered | 1.07 | 0.59—1.94 | 0.826 | 422 |  |  |  |  |
| Water treated by boiling | 0.51 | 0.25—1.03 | **0.062** | 459 | 0.46 | 0.21—0.98 | **0.044** |  |
| **Household socioeconomic variables** |  |  |  |  |  | | | |
| Has children under 5 in household | 0.71 | 0.40—1.25 | 0.233 | 464 |  |  |  |  |
| More than 6 people in household | 0.79 | 0.42—1.51 | 0.482 | 464 |  |  |  |  |
| Socioeconomic quintile^j^ |  |  |  | 464 |  |  |  | 464 |
| 3 | 0.17 | 0.07—0.41 | **<0.001** |  | 1.34 | 0.70—2.57 | 0.383 |  |
| 4 | 0.27 | 0.13—0.58 | **0.001** |  | 0.68 | 0.36—1.28 | 0.231 |  |
| 5 | 0.23 | 0.10—0.51 | **<0.001** |  | 0.53 | 0.28—1.01 | **0.055** |  |
| **Community-level environmental variables** |  |  |  |  |  | | | |
| Annual mean temperature^a^ | 1.14 | 0.91—1.43 | 0.264 | 464 |  |  |  |  |
| Annual max temperature^a^ | 1.18 | 0.91—1.51 | 0.205 | 464 |  |  |  |  |
| Annual min temperature^a^ | 1.11 | 0.90—1.36 | 0.320 | 464 |  |  |  |  |
| Mean temperature warmest quarter^a^ | 1.15 | 0.91—1.46 | 0.239 | 464 |  |  |  |  |
| Mean temperature coldest quarter^a^ | 1.12 | 0.91—1.38 | 0.291 | 464 |  |  |  |  |
| Temperature in hottest month^a^ | 1.19 | 0.93—1.53 | **0.174** | 464 | 1.31 | 0.71—2.44 | 0.386 | 464 |
| Temperature in coldest month^a^ | 1.09 | 0.90—1.32 | 0.360 | 464 |  |  |  |  |
| Temperature range^a^ | 1.03 | 0.60—1.75 | 0.927 | 464 |  |  |  |  |
| Slope^a^ | 0.97 | 0.90—1.06 | 0.517 | 464 |  |  |  |  |
| Elevation^a^ | 1.00 | 1.00—1.00 | 0.258 | 464 |  |  |  |  |
| EVI^a^ (factor of 100) | 1.05 | 0.96—1.15 | 0.286 | 464 |  |  |  |  |
| NDVI^a^ (factor of 100) | 0.99 | 0.91—1.07 | 0.806 | 464 |  |  |  |  |
| Monthly average precipitation^a^ | 0.99 | 0.97—1.01 | 0.436 | 464 |  |  |  |  |
| Mean precipitation in driest quarter^a^ | 0.98 | 0.88—1.09 | 0.770 | 464 |  |  |  |  |
| Mean precipitation in wettest quarter^a^ | 1.00 | 0.99—1.01 | 0.414 | 464 |  |  |  |  |
| Precipitation in driest month^a^ | 1.06 | 0.79—1.43 | 0.682 | 464 |  |  |  |  |
| Precipitation in wettest month^a^ | 1.00 | 0.99—1.00 | 0.377 | 464 |  |  |  |  |
| Land cover^k^ |  |  |  | 464 |  |  |  |  |
| Grassland | 0.15 | 0.01—1.74 | **0.131** |  | 0.79 | 0.02—34.15 | 0.902 |  |
| Savanna | 0.55 | 0.10—3.21 | 0.510 |  | 1.03 | 0.06—18.34 | 0.985 |  |
| Woody Savanna | 0.46 | 0.06—3.84 | 0.475 |  | 0.00 | 0.00—0.00 | 0.900 |  |
| Soil texture^l^ |  |  |  | 464 |  |  |  |  |
| Sandy Loam | 1.17 | 0.18—7.67 | 0.870 |  |  |  |  |  |
| Sandy Clay | 0.57 | 0.11—2.84 | 0.490 |  |  |  |  |  |
| Soil pH in 3 categories^m^ |  |  |  | 464 |  |  |  |  |
| neutral | 1.19 | 0.43—3.25 | 0.737 |  |  |  |  |  |
| basic | 1.00 | 0.16—6.31 | 0.998 |  |  |  |  |  |
| Soil pH in 5 categories^n^ |  |  |  | 464 |  |  |  |  |
| slightly acidic | 0.33 | 0.10—1.06 | **0.062** |  | 0.65 | 0.14—3.01 | 0.584 |  |
| neutral | 0.71 | 0.23—2.18 | 0.553 |  | 0.78 | 0.24—2.50 | 0.670 |  |
| slightly alkaline | 0.62 | 0.17—2.31 | 0.481 |  | 0.00 | 0.00—0.00 | 0.996 |  |

OR, odds ratio; aOR, odds ratio adjusting for age and sex; 95% CI, 95% confidence interval; No. of obvs., number of observations; EVI, enhanced vegetation index; NDVI, normalised difference vegetation index.

NDVI and EVI are indices quantifying vegetation and are measured between -1 to 1 with values closer to 1 indicating more vegetation. OR and aOR for NDVI refer to an 0.01 unit increase in NDVI as the variable was transformed by a factor of 100.

^a^Variable was encoded continuously. Reference categories are as follows: ^b^female; ^c^school has no toilet; ^d^no schooling; ^e^farmer; ^f^no toilet; ^g^surface water; ^h^water source less than 15 min; ^i^water always available or unavailable < 1 week/month; ^j^quintile 1 or 2; ^k^croplands; ^l^clay loam; ^m^acidic; ^n^moderately acidic.

**Supplementary Table S7.** Results of sensitivity analysis for Ascaris spp. multivariable logistic regression excluding environmental variables.

| Covariate | aOR | 95% CI | *P* value |
| --- | --- | --- | --- |
| Age (years)^a^ | 1.02 | 0.94—1.12 | 0.586 |
| Male sex^b^ | 0.82 | 0.52—1.28 | 0.386 |
| School toilet use^c^ |  |  |  |
| Does not use school toilet | **2.30** | **1.21—4.38** | **0.011** |
| Uses school toilet | 0.31 | 0.03—3.12 | 0.322 |
| Household water is treated by boiling^d^ | **0.29** | **0.13—0.67** | **0.004** |
| Random effects variance (95% CI) |  |  |  |
| School | 1.74(0.42—7.20) |  |  |
| Community | 0.27(0.043—1.72) |  |  |

aOr, adjusted odds ratio; 95% CI, 95% confidence interval.

*P* value is bold if *P* < 0.05.

Reference categories are as follows: ^a^continuous variable; ^b^female sex; ^c^school does not have toilet; ^d^household water is not treated by boiling.

The model includes 456 individuals from six schools and 17 communities, with school and community as random effects. Household water treatment refers only to boiling water as this was the only reported method.
